# Supplementary material for: Simultaneous EEG-fNIRS study of visual cognitive processing: ERP analysis and decision-related hemodynamic responses in healthy adults
Source: PLoS One. 2025 Jun 11;20(6):e0325017. doi: 10.1371/journal.pone.0325017 (PMC12157042; doi:10.1371/journal.pone.0325017)
Supplement: Attachment — Submitted filename: Response to Reviewers.docx [file pone.0325017.s002.docx]

**AUTHOR RESPONSES TO THE REVIEWERS**

**Journal:** PLOS ONE

**Title of the manuscript:** Simultaneous EEG-fNIRS Study of Visual Cognitive Processing: ERP Analysis and Decision-Related Hemodynamic Responses in Healthy Adults

**Manuscript ID Number**: PONE-D-25-18575

**Authors:** Tolga Turay, Onur Erdem Korkmaz, Ebru Ergün

We would like to start by thanking the editor and the reviewer for their useful comments and suggestions on how to further improve the quality of our manuscript. We have been able to incorporate changes to reflect all the suggestions provided by the reviewer. Here is a point-by-point response to the reviewer’s comments and concerns.

**Reviewer 1:**

**1. Asked:** “The abstract is mostly clear; however, the sentence beginning with "Although descriptive variability in HbO..." should be rephrased for better clarity and flow.”

**1. Answer:** We thank the reviewer for this valuable suggestion. In response, we have revised the sentence to improve clarity and readability. The updated version now reads: *“While visual inspection revealed variability in oxygenated hemoglobin (HbO) levels across channels and conditions, statistical analyses using Cohen’s D and one-way ANOVA did not identify any significant differences between the conditions (p > 0.05).”*

**2. Asked:** “A diagram or flowchart summarizing the fNIRS preprocessing pipeline would significantly improve clarity for readers unfamiliar with the multi-step procedure”

**2. Answer:** We appreciate the reviewer’s insightful suggestion to improve the clarity of our methodology. In response, we have added a new visual summary illustrating the main steps of the fNIRS preprocessing pipeline. This diagram is now included as Figure 3 in the revised manuscript and provides an overview of the key stages from raw data conversion to statistical analysis.

**3. Asked:** “In the Results section, Tables 3 and 4 could benefit from deeper commentary. Consider walking through a representative channel (e.g., PO4) in more detail.”

**3. Answer:** We thank the reviewer for this valuable suggestion. In response, we added a specific commentary highlighting the PO4 channel after the description of Table 3. This additional note emphasizes the enhanced wavelet power observed in the theta and low alpha bands for the RR and RF conditions, providing a representative example of the observed patterns.

**4. Asked:** “In the Discussion, the non-significance of fNIRS results should be explored more thoroughly. Possible explanations may include inter-individual variability, low signal-to-noise ratio, or spatial resolution limitations.”

**4. Answer:** We thank the reviewer for this important suggestion. In response, we have expanded the Conclusion section by adding a sentence that addresses potential reasons for the lack of significant fNIRS findings, including inter-individual variability, low signal-to-noise ratio, and spatial resolution limitations. This addition follows the initial interpretation of the non-significant HbO results.

**5. Asked:** “There are some stylistic redundancies throughout the manuscript (e.g., repeated definitions of “motivation” and “ERP”). These could be condensed to enhance readability.”

**5. Answer:** We thank the reviewer for this valuable observation. In response, the manuscript has been carefully reviewed and revised to eliminate stylistic redundancies, including repeated definitions of key concepts such as “motivation” and “ERP,” thereby improving overall readability and flow.

**6. Asked:** “Some sentences are overly long or passive in structure. Refining the language for clarity and conciseness would improve the paper’s flow.”

**6. Answer:** We thank the reviewer for this constructive feedback. Accordingly, the manuscript has been thoroughly revised to improve clarity and conciseness by refining overly long or passive sentences, thereby enhancing the overall flow of the text.

**7. Asked:** “Clarification of EEG and fNIRS Channel Locations (Figure 2) In Figure 2, EEG and fNIRS sensors are plotted simultaneously; however, it remains unclear which markers correspond to EEG electrodes and which to fNIRS optodes. Please clearly distinguish them either through labels, shapes, or a color legend. I also recommend updating the figure caption to explicitly indicate which symbols represent EEG and fNIRS channels. Suggested caption addition: "EEG electrodes are shown as [color/symbol], whereas fNIRS optodes (sources and detectors) are shown as [color/symbol].”

**7. Answer:** We thank the reviewer for this important observation. In response, we have updated Figure 2 by adding a clear legend and labels to distinguish EEG electrodes from fNIRS sources and detectors. The figure caption has also been revised to explicitly describe the markers representing EEG and fNIRS components.

**8. Asked:** “Missing Formula for Cohen’s d (Figure 4) Cohen’s d is used to generate Figure 4, but the manuscript does not state the formula used for its computation. Please include the formula or a clear citation to ensure reproducibility.”

**8. Answer:** We thank the reviewer for this valuable suggestion. In response, we have added the explicit formula used for the computation of Cohen’s d in the revised manuscript to enhance clarity and reproducibility. A detailed explanation of the parameters involved in the formula has also been provided.

**9. Asked:** “Inconsistent Y-Axis Ranges in Table 4 The subplots in Table 4 appear to use varying y-axis scales, which hinders visual comparison between conditions. I recommend standardizing the y-axis (e.g., from –10 to 10) across all plots unless variability is justified and explained in the caption. Suggested note: "All y-axes were scaled uniformly from –10 to 10 µV for consistent comparison.”

**9. Answer:** We thank the reviewer for this important suggestion. In response, Table 4 has been revised, and the y-axis ranges across all subplots have been standardized to allow for consistent and meaningful comparisons between conditions. This update has substantially improved the clarity of the figure.

**10. Asked:** “Missing Descriptive Statistics for Hemodynamic Responses (Table 4) Table 4 summarizes fNIRS responses but lacks descriptive statistics such as means and variances. Including a supplementary table that reports mean ± standard deviation values would enhance transparency and allow for deeper interpretation of the hemodynamic data. Suggested improvement: "A summary table reporting the mean and variance of HbO/HbR values per condition would strengthen the results.”

**10. Answer:** We thank the reviewer for this valuable suggestion. In response, a new supplementary table (Table 5) has been created, presenting the mean ± standard deviation values for each channel and stimulus condition. This addition enhances the transparency of the fNIRS results and facilitates deeper interpretation of the hemodynamic patterns.

**11. Asked:** “Inconsistent Image Ordering in Table 3 In Table 3, the order of images across rows is inconsistent, which may confuse readers. Ensure that the same ordering of visual elements (conditions, time points, or regions) is maintained across all rows for clarity.

Suggested instruction: "Use a fixed left-to-right ordering of conditions across all rows (e.g., [A, B, C]).”

**11. Answer:** We thank the reviewer for this helpful observation. In response, the inconsistency in the ordering of images in Table 3 has been corrected, and a revised version with a fixed and uniform order across all rows has been included in the manuscript to improve clarity and coherence.

**Reviewer 2:**

**1. Asked:** “The simultaneous plotting of EEG electrodes and fNIRS optodes in Figure 2 is currently unclear. It is difficult for the reader to distinguish which symbols correspond to EEG and which to fNIRS components. Please revise the figure by using different shapes and/or colors for EEG electrodes and fNIRS sources/detectors, and update the figure caption accordingly. This distinction is crucial for understanding the multimodal setup.”

**1. Answer:** We appreciate the reviewer’s observation regarding the clarity of sensor representation in Figure 2. In response, the figure has been revised to visually differentiate EEG electrodes from fNIRS sources and detectors using distinct colors and marker shapes. The figure caption has also been updated to explicitly define each symbol, ensuring that the multimodal setup is easily interpretable.

**2. Asked:** “While the fNIRS preprocessing pipeline is described in detail, readers unfamiliar with fNIRS analysis might find it difficult to follow the multiple steps. Including a flowchart or diagram summarizing the preprocessing steps (motion correction, filtering, PCA, epoching, etc.) would substantially enhance the clarity and accessibility of this section.”

**2. Answer:** We thank the reviewer for highlighting the need to improve the accessibility of the preprocessing description. To address this, we have added a flowchart (Figure 3) that visually outlines the major steps of the fNIRS preprocessing pipeline. This figure is intended to assist readers—particularly those less familiar with fNIRS methodology in understanding the sequential structure of the analysis process.

**3. Asked:** “Cohen’s d values were used extensively, especially in interpreting fNIRS results (e.g., Figure 4), but the exact formula for Cohen’s d calculation is not provided. Please explicitly state the formula or provide a precise reference. Reproducibility is a key requirement.”

**3. Answer:** We thank the reviewer for emphasizing the importance of transparency in reporting effect size metrics. In response, we have explicitly included the formula used to compute Cohen’s d in the revised manuscript, along with a clear explanation of the corresponding variables. This addition aims to ensure full reproducibility and clarity of the analysis.

**4. Asked:** “Subplots in Table 4 (fNIRS responses) have inconsistent y-axis ranges across conditions. This inconsistency complicates visual comparison. Standardizing the y-axis scale across all subplots (e.g., –10 to 10 µV for EEG plots, appropriate range for HbO concentration in fNIRS) would improve interpretability. If variability requires separate scaling, this should be justified clearly in the figure captions.”

**4. Answer:** We appreciate the reviewer’s helpful feedback regarding the visual consistency of Table 4. To address this issue, we have standardized the y-axis ranges across all subplots to facilitate clearer comparisons between conditions. This adjustment improves the interpretability of the data and ensures a more coherent visual presentation.

**5. Asked:** “Table 4 presents averaged fNIRS hemodynamic responses but lacks descriptive statistics (mean ± standard deviation) across participants. This omission reduces the transparency and interpretability of the findings. Please add a supplementary table including means and variances for each condition and channel.”

**5. Answer:** We are grateful to the reviewer for pointing out this important omission. To improve the transparency and interpretability of our results, we have created an additional table (Table 5) that presents the mean and standard deviation values of HbO responses for each condition and channel. This supplementary table complements Table 4 and provides a more detailed quantitative summary of the hemodynamic data.
